# Primary immunodeficiency as a cause of immune-mediated kidney diseases

**DOI:** 10.1093/ndt/gfae117

**Published:** 2024-05-21

**Authors:** Changming Zhang, Dandan Liang, Zhihong Liu

**Affiliations:** National Clinical Research Center for Kidney Diseases, Jinling Hospital, Affiliated Hospital of Medical School, Nanjing University, Nanjing, China; National Clinical Research Center for Kidney Diseases, Jinling Hospital, Affiliated Hospital of Medical School, Nanjing University, Nanjing, China; National Clinical Research Center for Kidney Diseases, Jinling Hospital, Affiliated Hospital of Medical School, Nanjing University, Nanjing, China

**Keywords:** autoimmunity, complement mediated kidney diseases, diagnosis and treatment, lupus nephritis, primary immunodeficiency

## Abstract

Primary immunodeficiency (PID) is no longer defined by infections alone, and autoimmunity is an accompanying manifestation of PID. Recurrent infections may trigger autoimmunity through molecular mimicry, bystander activation or superantigens. The diagnosis of PID is still challenging, but genetic analysis reveals the underlying link between PID and autoimmunity. Mutations in relevant genes affecting central and peripheral immune tolerance, regulatory T-cell function, expansion of autoreactive lymphocytes, antigen clearance, hyperactivation of type I interferon and nuclear factor-κB pathways have all been implicated in triggering autoimmunity in PID. Autoimmunity in PID leads to chronic inflammation, tissue damage and organ failure, and increases the mortality of patients with PID. The kidneys are inextricably linked with the immune system, and kidney diseases can be mediated by both infection and autoimmunity/inflammation in PID patients. The manifestations of kidney involvement in PID patients are very heterogeneous and include lupus nephritis, C3 glomerulopathy, kidney thrombotic microangiopathy, vasculitis and interstitial nephritis. Patients with PID-caused kidney diseases have defined immune function defects and may benefit from pathway-based biologics, stem cell transplantation or gene therapy. Early diagnosis and appropriate treatment of PID are crucial for reducing the mortality rate and improving organ function and quality of life.

## INTRODUCTION

Primary immunodeficiency (PID), also referred to as inborn errors of immunity, comprises a heterogeneous group of disorders impairing the development, regulation and function of the immune system [1]. PID not only presents with an increased susceptibility to infections, but is also prone to immune dysregulation, such as autoimmunity, autoinflammation, allergy and/or malignancy [2–4]. PID is caused mainly by monogenic gene mutations that affect the immune system. With the advancement of genome-sequencing technologies, more than 450 genes that cause different PIDs have been identified. PIDs are divided into 10 categories according to their underlying molecular defects by the International Union of Immunodeficiency Societies (IUIS) [1]. Because of the variability and heterogeneity of the manifestations of PID, diagnosis and treatment can be delayed. Additionally, autoimmune/inflammatory processes occur throughout the patient's lifetime and have prognostic significance. Overall survival time was significantly shorter in patients with PID accompanied by autoimmune/inflammatory diseases than in those without these manifestations [5].

The kidneys are inextricably linked with the immune system. Kidney diseases can be mediated by both infection and autoimmunity/inflammation and have been reported in almost all PID categories [6]. In this review, we focus on the pathogenesis of PID-caused kidney diseases, disease diagnosis and recent emerging therapies for PID-caused kidney diseases.

### Primary immunodeficiency

PID can be divided into two forms. Monogenic PIDs are caused by mutations in genes involved in the development and the function of the immune system. Polygenic immunodeficiencies are characterized by a heterogeneous clinical presentation and may have a multifactorial etiology [3]. Although infection is a hallmark feature of both forms of PID, both forms also frequently present with autoimmunity and autoinflammation [2–4]. Autoimmunity/inflammation was observed in 26% of patients with PID. This is a much greater percentage of patients, with at least 10-fold greater incidence than that in the general population [5]. Unlike in the general population, autoimmunity in PID does not have an age or sex predominance. Almost the whole spectrum of autoimmune diseases, such as autoimmune cytopenia, inflammatory bowel disease, interstitial lung disease, autoimmune thyroid disease, type 1 diabetes mellitus and rheumatologic disorders, can be observed in patients with PID. The wide range of autoimmune/inflammatory manifestations indicates the complexity of underlying mechanisms and diverse clinical syndromes.

The exact mechanism of autoimmunity in PID is not clear. Recurrent infections may trigger autoimmunity through several mechanisms: (i) molecular mimicry: infectious pathogens may share similarities with human proteome initiating a phenomenon of cross-reactivity; (ii) bystander activation: frequent and recurrent infectious diseases induce an over-production of proinflammatory cytokines such as interleukin (IL)-23 which help to differentiate autoreactive T-cell subsets [e.g. T-helper 17 (Th17)]; (iii) infectious diseases may induce cell damage and “cryptic antigens” exposure which will be presented by macrophages to autoreactive T cells; and (iv) superantigens produced by infectious agents can induce a nonspecific activation of autoreactive T cells. Genetic analysis has established another link between PID and autoimmunity because genetic mutations may affect the activity of multiple cell types or signaling molecules involved in triggering autoimmunity. Gene mutations affecting central and peripheral immune tolerance, regulatory T-cell (Treg cell) function, expansion of autoreactive lymphocytes, antigen clearance, hyperactivation of type I interferon and nuclear factor (NF)-κB pathways have all been implicated in triggering autoimmunity in PID [3, 7]. Autoimmunity in PID leads to chronic inflammation, tissue damage and organ failure, and increases the mortality of patients with PID.

Kidney involvement is relatively rare in PID. However, compared with that in the general population, the risk of immune-mediated kidney disease in PID is increased 8-fold. The kidney manifestations in patients with PID may include recurrent and persistent hematuria, nephrotic syndrome and rapidly progressive glomerulonephritis [5]. Lupus nephritis is common in PID and is caused by mutations in the type I interferon, NF-κB, JAK/STAT and phosphoinositide 3-kinase pathways [8]. These genes are also closely associated with primary immune dysregulation disorders, which are associated with the highest risk of autoimmunity in PID [9]. In common variable immunodeficiency, the most common PID, kidney diseases affect approximately 2% of patients. The main form of kidney disease is immune complex glomerulonephritis, followed by tubulointerstitial nephritis [10]. Light-chain amyloidosis and pauci-immune glomerulonephritis have also been reported [10]. Other types of PID, such as type I interferonopathy, within the broader classification of autoinflammatory diseases, and complement deficiency, may also indicate kidney diseases, which will be described in more detail below [6, 11].

### Mechanism of PID-caused kidney diseases

**Figure 1:**
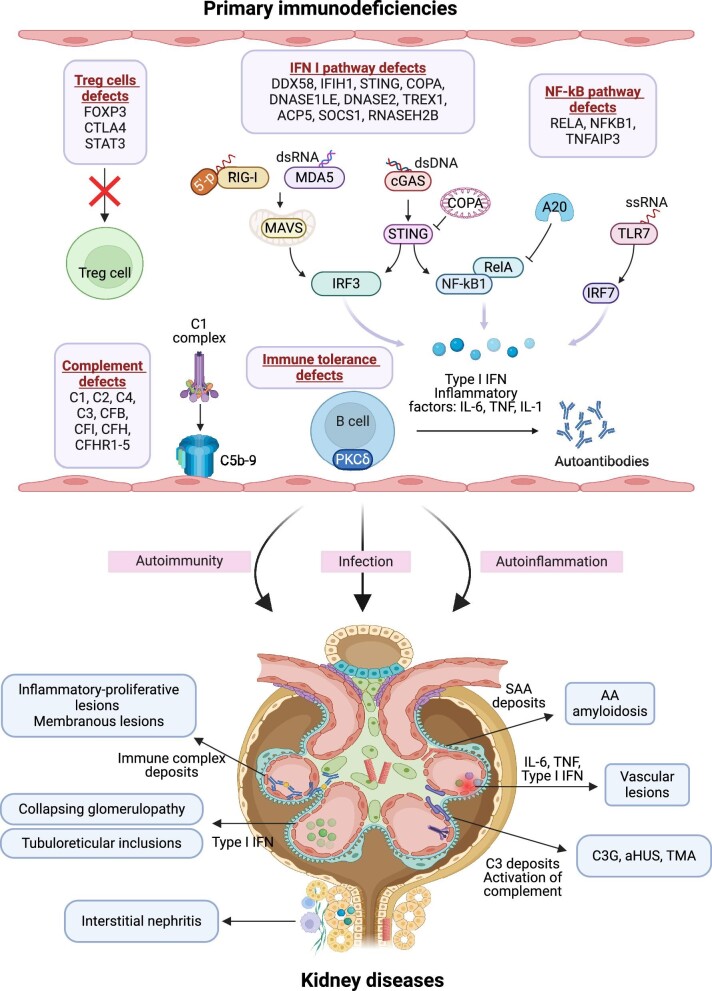
Mechanism of PID-caused kidney diseases. Genetic defects in B-cell tolerance, Treg cells, type I interferon, TLR7, NF-κB and complement pathways associated with PID-caused kidney diseases are shown. *FOXP3, CTLA4* and *STAT3* mutations affect Treg cell function. RIG-I/MDA5 and cGAS recognize dsRNA and dsDNA, respectively. The type I interferon pathway is subsequently activated through MAVS or STING signal transduction. NF-κB signaling is also activated by STING. A20 negatively regulates NF-κB signaling. TLR7 senses ssRNA and activates type I interferon signaling. Abbreviations: FOXP3, forkhead box P3; CTLA4, cytotoxic T-lymphocyte associated protein 4; STAT3, signal transducers and activators of transcription 3; DNASE1L3, deoxyribonuclease1-like 3; TREX1, three prime repair exonuclease 1; dsRNA, double-stranded RNA; dsDNA, double-stranded DNA; RIG-I, retinoic acid-inducible gene I; MDA5, melanoma differentiation-associated protein 5; cGAS, cyclic GMP-AMP synthase; MAVS, mitochondrial antiviral signaling protein; STING, stimulator of interferon genes; IRF, interferon regulatory factor; IFN, interferon; COPA, COPI coat complex subunit alpha; ACP5, acid phosphatase 5, tartrate resistant; DNASE2, deoxyribonuclease2; ssRNA, single-stranded RNA; TLR7, toll-like receptor 7; NF-κB, nuclear factor kappa B; RelA, RELA proto-oncogene, NF-κB subunit; SOCS1, suppressor of cytokine signaling 1; PKCδ, protein kinase C δ; aHUS, atypical hemolytic–uremic syndrome; TMA, thrombotic microangiopathy; C3G, C3 glomerulopathy. Created with BioRender.com.

In patients with PID, the loss of immune tolerance is obvious. Once immune tolerance is lost, multiple interconnected innate and adaptive immune processes may lead to autoimmune diseases and mediate organ damage, including kidney damage. The probable mechanisms of PID-caused kidney diseases are described below (Fig. 1).

#### Genetic defects in *PRKCD* and kidney diseases

Protein kinase C δ (PKCδ), encoded by the *PRKCD* gene, is a serine/threonine kinase that has been implicated in the control of B-cell homeostasis and tolerance. The key features of PKCδ deficiency are autoimmunity, lymphoproliferation and susceptibility to infection [12]. Many cases presented with features of systemic lupus erythematosus (SLE). PKCδ deficiency was described as a genetic cause of SLE and lupus nephritis since 2013 [13]. Five patients with kidney manifestations have been reported, with one kindred carrying the G510S biallelic mutation particularly affected [12]. Lupus nephritis was described in three cases, while membranous nephropathy and nephrotic syndrome was described in one case [13, 14]. In addition, SLE and tubular interstitial nephritis was described in one case, who was treated with ofatumumab, a humanized anti-CD19 monoclonal antibody, and showed marked clinical improvement [15, 16].

#### Genetic defects in Treg cell function and kidney diseases

Forkhead box P3 (FOXP3) and cytotoxic T-lymphocyte associated protein 4 (CTLA4) are master regulators of Treg cells and are responsible for regulating peripheral tolerance by inhibiting the activation of T lymphocytes. Mutations in FOXP3 impair Treg cells and cause immune dysregulation polyendocrinopathy enteropathy X-linked (IPEX) syndrome. Up to 25% of patients with FOXP3 mutations develop kidney diseases, including interstitial nephropathy and membranous glomerulonephritis [17], and kidney involvement could be the first manifestation of the disease. Patients with CTLA4 haploinsufficiency present with autoimmune cytopenias, enteropathy, interstitial lung disease, extra-lymphoid lymphocytic infiltration and recurrent infections. Kidney involvement has been rarely reported, with only one patient with granulomatous interstitial nephritis [18]. Gain-of-function (GOF) mutations in signal transducer and activator of transcription 3 (STAT3) cause reduced Treg cells. Patients with STAT3 GOF mutations are characterized by early-onset lymphoproliferation, autoimmunity, growth delay and recurrent infections. Approximately 5% of such patients develop immune-mediated glomerulonephritis [19]. These studies indicated the important role of Treg cells in kidney involvement caused by PID, and Treg cell-based therapy may benefit such patients [20].

#### Genetic defects in the type I interferon pathway and lupus nephritis or collapsing glomerulopathy

Type I interferons are produced by the recognition of nucleic acids by endosomal or cytosolic sensors [toll-like receptors, cyclic GMP-AMP synthase (cGAS), melanoma differentiation-associated protein 5 (MDA5) or retinoic-acid-inducible gene I (RIG-I)]. Then, through different signaling pathways and adaptor molecules, interferon regulatory factors or stimulator of interferon-genes (STING) is activated, which drives interferon transcription. Once released, type I interferon binds to heterodimeric interferon-α/β receptor 1/2 (IFNAR1/2), resulting in the phosphorylation of Janus kinase 1 (JAK1) and TYK2 and subsequent activation of the transcription factor complex ISGF3. ISGF3 binds to interferon-stimulated response elements in gene promoters and induces the expression of interferon-stimulated genes (ISGs). USP18 and the ubiquitin-like protein ISG15 are negative regulators of downstream signaling via IFNAR1/2. Mutations in any of these proteins activate type I interferon signaling and cause type I interferonopathy.

Type I interferons can cause kidney diseases through direct damage to intrinsic kidney cells [21]. Interferon mediates endothelial dysfunction, promotes mesangial cell proliferation, elevates the ISGs expression in podocytes, and induces podocyte apoptosis, podocin derangement and albumin filtration [22]. Type I interferons can indirectly cause kidney damage by triggering immune responses. Interferon promotes T-cell priming and activation by enhancing MHC II expression, antigen presentation, dendritic cell maturation, costimulatory molecules expression, and recruitment of monocytes and natural killer (NK) cells [23, 24]. Interferon also induces the expression of B-cell-activating factor (BAFF) in monocytes [25], which is required for B-cell maturation and antibody production [26–28]. Recruitment of macrophages in the presence of interferon-α also promotes nephritis [29]. Together, these events promote generation of nuclear antigens and autoantibodies and enhance the formation of immune complexes.

Lupus nephritis is frequently reported in type I interferonopathy. Loss-of-function (LOF) variants in deoxyribonuclease 2 (*DNASE2*) and three-prime repair exonuclease 1 (*TREX1*) result in defective nucleic acid degradation, causing Aicardi–Goutières syndrome, SLE and familial chilblain lupus. Lupus nephritis and kidney thrombotic microangiopathy (TMA) have been reported in such patients [30–32]. GOF mutations in *DDX58* cause lupus nephritis by reducing autoinhibition, which leads to RIG-I hyperactivation, increased RIG-I K63 ubiquitination and MAVS aggregation [33]. Heterozygous mutations in COPI coat complex subunit alpha (*COPA*) gene cause enhanced trafficking of STING to the Golgi and aberrant activation of the type I interferon pathway [34]. Patients with *COPA* mutations present with autoimmune inflammatory arthritis and interstitial lung disease with Th17 dysregulation and autoantibody production. GOF mutations in *STING* underlie a type I interferonopathy termed STING-associated vasculopathy with onset in infancy (SAVI), which is variably characterized by early-onset systemic inflammation, skin vasculopathy and interstitial lung disease. Antineutrophil cytoplasmic antibody-associated vasculitis-like or lupus-like glomerulonephritis is commonly reported in both diseases. Biallelic mutations in the *ACP5* gene cause a rare disease named spondyloenchondro-dysplasia with immune dysregulation (SPENCD) characterized by short stature, spastic paraparesis, intracranial calcification and immune dysregulation. Approximately 85% of patients develop autoimmune diseases, including autoimmune thrombocytopenia (46%), SLE (36%) and lupus nephritis (26%) [35]. Affected individuals exhibit an absence of serum tartrate-resistant acid phosphatase, elevated levels of serum interferon-α and an upregulation of ISGs in whole blood. Suppressor of cytokine signaling 1 (SOCS1) negatively regulates type I and type II interferon signaling by inhibiting the JAK/STAT pathway. Patients with SOCS1 haploinsufficiency are characterized by early-onset multisystemic autoimmunity (immune thrombocytopenic purpura, autoimmune hemolytic anemia, SLE, psoriasis, thyroiditis, hepatitis), lymphoproliferation and recurrent bacterial infections. Lupus nephritis has been reported in three patients [36, 37].

Collapsing glomerulopathy is a glomerular injury pattern characterized by global or segmental collapse of the glomerular tuft, with capillary wall wrinkling and hyperplasia of parietal epithelial cells (PECs) migrating to the tuft to generate pseudocrescents. Collapsing glomerulopathy is linked to viral infections, autoimmune diseases, APOL1 risk variants and interferon therapy. A patient of collapsing glomerulopathy with 2 APOL1 risk alleles and endogenous overproduction of type 1 interferon secondary to SAVI has been reported [38]. Additionally, collapsing glomerulopathy was reported in a patient with Aicardi–Goutières syndrome secondary to a *RNASEH2B* mutation. The patient's biopsy showed type I interferon induced signals in PECs, whose dysregulation is involved in the pathogenesis of collapsing glomerulopathy [39].

#### Genetic defects in Toll-like receptor 7 signaling and lupus nephritis

Toll-like receptor 7 (TLR7) is a pattern recognition receptor located in endosomes of immune cells and can detect viral and bacterial single-stranded RNA. TLR7 stimulation generally activates the NF-κB, type I interferon and mitogen-activated protein kinase pathways, and triggers the transcription of inflammatory cytokines, chemokines and costimulatory molecules. An increase in TLR7 exacerbates autoimmunity and nephritis in mice. Genome-wide association studies have shown that polymorphisms in TLR7 are associated with lupus risk and cause more pronounced interferon signatures in humans. TLR7 allows the persistent activation of dendritic cells and B cells by autoantigens, thereby promoting autoantibody production and glomerulonephritis mediated by immune complexes. In addition, the nucleic acid component of immune complexes activates intrarenal macrophages via TLR7, resulting in the activation of glomerular endothelium and mesangial cells in lupus nephritis [40]. These studies support a role for TLR7 activation in the pathogenesis of lupus nephritis.

A GOF *de novo* mutation in *TLR7* has been shown to cause human SLE and lupus nephritis [41]. The TLR7^Y264H^ variant selectively increased the sensing of guanosine and 2′,3′-cGMP. Introduction of this variant into mice was sufficient to drive lupus nephritis. Enhanced TLR7 signaling drives aberrant accumulation of age-associated B cells and the production of autoantibodies. Deficiency of MyD88 (an adaptor protein downstream of TLR7) rescued autoimmunity, aberrant B-cell survival, and all cellular and serological phenotypes.

#### Genetic defects in the NF-κB pathway and lupus nephritis

The NF-κB family has five members: p65 (RelA, encoded by *RELA*), RelB, c-Rel, NF-κB1 (p50, encoded by *NFKB1*) and NF-κB2 (p52). They combine to form hetero or homodimers and bind to DNA. The NF-κB protein is inactive in the cytoplasm through binding to the inhibitory protein IκB. Upon exposure to stimuli, extracellular and intracellular receptors trigger signal transduction events that lead to activation of the IκB kinase (IKK) complex. Once activated, IKKβ phosphorylates IκBs for degradation. NF-κB is subsequently released into the nucleus, where NF-κB-mediated transcriptional activation occurs. Genetic defects in crucial components of the NF-κB pathway that underlie various clinical phenotypes in humans, such as immunodeficiency, autoimmunity and SLE, have been identified by genome sequencing.

Heterozygous LOF mutations in *RELA* cause chronic mucocutaneous ulceration and autoimmune lymphoproliferative syndrome (ALPS). Heterozygous LOF mutations in *NFKB1* cause recurrent sinopulmonary infections, lymphoproliferation and autoimmunity. *RELA* and *NFKB1* mutations have been identified in patients with lupus nephritis [8]. These patients exhibited pronounced autoimmunity, with multiple autoantibodies, autoimmune hemolytic anemia, thrombocytopenia and massive immune complex deposits in kidneys. They also presented with lymphoproliferative diseases and severe infections, indicating multiple immune dysregulation events in these patients.

Deubiquitination plays an important role in the regulation of NF-κB activity. A20 is a deubiquitinating enzyme encoded by *TNFAIP3*. It deubiquitinates key signaling molecules, such as receptor-interacting serine/threonine-protein kinase 1 (RIPK1) and NF-κB essential modulator (NEMO), and negatively regulates the activity of NF-κB pathway. Heterozygous LOF mutations in *TNFAIP3* cause an early-onset autoinflammatory disease resembling Behçet's disease [42]. Three novel *TNFAIP3* mutations have been reported as causes of lupus nephritis, adding *TNFAIP3* mutations to the list of causes of Mendelian lupus nephritis [43]. These patients exhibited significant activation of the NF-κB and type I interferon pathways.

#### Complement deficiency and lupus nephritis

The complement system contains three distinct enzymatic cascade pathways, namely the classical, alternative and lectin pathways. All of these pathways converge toward the cleavage of central C3 by a C3 convertase. This is followed by the formation of a C5 convertase, which cleaves C5 into C5a and C5b. This results in the formation of a membrane attack complex (MAC), which lyses the cell membrane and leads to the release of biologically active fragments that enhance inflammation, recruit leukocytes and promote host defense. These proinflammatory cascades require strict control by a range of soluble and membrane-bound regulatory proteins that act to limit complement-mediated damage to the host [44].

Early complement components facilitate the clearance of immune complexes and apoptotic cells. In patients with C1q deficiency, immune complexes preferentially engage plasmacytoid dendritic cells, resulting in the generation of interferon-α. Autoantigens presented by conventional dendritic cells in the presence of interferon-α promote the activation of autoreactive effector T cells and the development of autoimmunity [45]. Enhanced neutrophil extracellular trap formation is another factor that promotes autoimmunity in C1r deficiency [46]. Complement genetic defects are the strongest genetic factor for lupus. Approximatly 65%–88% of patients with C1 deficiency develop lupus, and 30%–40% of those patients develop glomerulonephritis [44].

#### Complement deficiency and C3 glomerulopathy/TMA

Genetic defects are observed in 25% of patients with C3 glomerulopathy. Pathogenic variants in C3, complement factor B (CFB) and complement factor I (CFI) have been reported. These mutations resulted in hyperactive C3 convertase, and increased C3b deposition on cell surfaces indicating the activation of alternative complement pathway [47].

Sequence and copy number variations in the *CFHR* gene cluster (*CFH, CFHR1, CFHR2, CFHR3, CFHR4* and *CFHR5*) are linked to atypical hemolytic uremic syndrome (aHUS) and C3 glomerulopathy [48]. Distinct genetic alterations, deletions or duplications generate hybrid or mutant *CFHR* genes, as well as hybrid *CFHR*–*CFH* genes, and alter the CFHR and CFH plasma repertoire. *CFHR1, CFHR3* and *CFH* gene alterations combined with intact *CFHR2, CFHR4* and *CFHR5* genes cause aHUS. Alterations in each of the five *CFHR* genes combined with an intact *CFH* gene cause C3 glomerulopathy [48].

Pathogenic or likely pathogenic variants in complement genes have also been identified in patients with TMA, especially in those with aHUS-associated TMA. The variants were located mainly in CFH, CFI and membrane cofactor proteins (MCP). In addition, rare variants or polymorphisms in *CFH, CFI, CFB, MCP, thrombomodulin, plasminogen* and *CFHR* genes were detected in 6 of 10 patients with lupus nephritis-associated TMA [49].

#### Genetic defects of *SMARCAL1* and kidney diseases

Biallelic mutations in *SMARCAL1*, which encodes a DNA annealing helicase with roles in DNA replication fork restart, DNA repair and gene expression modulation, cause Schimke immuno-osseous dysplasia (SIOD), an ultrarare disease characterized by short stature with spondyloepiphyseal dysplasia, T-cell immunodeficiency and progressive nephropathy. Nearly all affected individuals have steroid-resistant nephropathy, usually evolving into end-stage kidney disease. The predominant renal pathology has been reported as focal segmental glomerulosclerosis in 83% of individuals [50].

#### PID and AA amyloidosis in kidneys

AA amyloidosis is a common and severe glomerular presentation of several PIDs, especially autoinflammatory diseases. Recently, we have reviewed the mechanism and clinical manifestations of kidney AA amyloidosis in patients with autoinflammatory diseases [6]. In the present review, we listed these PIDs in Table 1.

**Table 1:** PID-caused kidney diseases.

Diseases^a^	Genetic defect	Inheritance	Mechanism	Key clinical features^a^	Kidney manifestations
Defects of B-cell tolerance					
PRKCD deficiency	PRKCD	AR LOF	Apoptotic defect in B cells	SLE-like autoimmunity, recurrent infections, lymphoproliferation	Lupus nephritis, membranous nephropathy, tubular interstitial nephritis
Defects of Treg cells					
IPEX	FOXP3	XLR LOF	Impaired Tregs function	Autoimmune enteropathy, early-onset diabetes, thyroiditis, hemolytic anemia, thrombocytopenia, eczema, elevated IgE and IgA	Membranous glomerulonephritis, interstitial nephritis
CTLA4 haploinsufficiency	CTLA4	AD LOF	Impaired Tregs function	Autoimmune cytopenias, enteropathy, interstitial lung disease, extra-lymphoid lymphocytic infiltration, recurrent infections	Granulomatous interstitial nephritis
STAT3 GOF	STAT3	AD GOF	Decreased Tregs and impaired function	Lymphoproliferation, solid organ autoimmunity, recurrent infections	Immune-mediated glomerulonephritis
Defects of type I interferon pathway					
DNASE1L3 deficiency	DNASE1L3	AR LOF	Defect dsDNA clearance, activation of IFN-I pathway	Very early-onset SLE, reduced complement levels, autoantibodies (dsDNA, ANCA), hypocomplementemic urticarial vasculitis syndrome	Lupus nephritis, thrombotic microangiopathy
DNAse II deficiency	DNASE2	AR LOF	Defect DNA clearance, activation of IFN-I pathway	AGS, autoinflammatory pancytopenia syndrome, SLE	Lupus nephritis
TREX1 deficiency	TREX1	AR, AD LOF	Defect DNA clearance, activation of IFN-I pathway; or Free glycan↑	Classical AGS, SLE, retinal vasculopathy with cerebral leukoencephalopathy and systemic manifestations, FCL	Lupus nephritis, thrombotic microangiopathy
AGS7	IFIH1	AD GOF	Enhanced dsRNA sensing, activation of IFN-I pathway	Classical AGS, SLE, spastic paraparesis, Singleton-Merten syndrome	Lupus nephritis
GRAD	DDX58	AD GOF	Enhanced dsRNA sensing, activation of IFN-I pathway	Singleton-Merten syndrome, SLE	Lupus nephritis
SAVI	STING	AD GOF	Enhanced DNA sensing, activation of IFN-I pathway	Skin vasculopathy, inflammatory lung disease, systemic autoinflammation and intracranial calcification, FCL, SLE	Lupus nephritis, thrombotic microangiopathy, collapsing glomerulopathy
COPA defect	COPA	AD LOF	Trafficking of STING↑, activation of IFN-I pathway	Autoimmune inflammatory arthritis and interstitial lung disease with Th17 dysregulation and autoantibody production, SLE	Lupus nephritis
SPENCD	ACP5 (TRAP)	AR LOF	Defect TRAP, activation of IFN-I pathway	Short stature, spastic paraparesis, intracranial calcification, SLE, thrombocytopenia and autoimmune hemolytic anemia, possibly recurrent bacterial and viral infections	Lupus nephritis
SOCS1 haploinsufficiency	SOCS1	AD LOF	↑pSTAT1, ↑IFN-I/II signature	Early-onset severe multisystemic autoimmunity, neutropenia, lymphopenia, ITP, autoimmune hemolytic anemia, SLE, hepatosplenomegaly, psoriasis, arthritis, thyroiditis, hepatitis; recurrent bacterial infections	Lupus nephritis
RNASEH2B deficiency, AGS2	RNASEH2B	AR LOF	Intracellular accumulation of abnormal RNA–DNA hybrid species, activation of IFN-I pathway	Classical AGS, spastic paraparesis	Collapsing glomerulopathy
Defects of Toll-like receptor 7 signaling					
SLE17^b^	TLR7	XLD GOF	Activation of TLR7 pathway	SLE	Lupus nephritis
Defects of NF-κB pathway					
NFKB1 deficiency	NFKB1	AD LOF	Dysregulation of NF-κB pathway	Recurrent sinopulmonary infections, COPD, lymphoproliferation, autoimmune cytopenias and autoimmune thyroiditis	Glomerulonephritis, lupus nephritis
RelA haploinsufficiency	RELA	AD LOF	Dysregulation of NF-κB pathway	Chronic mucocutaneous ulceration, ALPS, SLE	Lupus nephritis
A20 haploinsufficiency	TNFAIP3	AD LOF	Defects of deubiquitination, activation of NF-κB pathway	Arthralgia, mucosal ulcers, ocular inflammation, SLE, PFAPA, IBD	Lupus nephritis
Complement deficiency					
C1 deficiency	C1QA, C1QB, C1QC, C1R, C1S	AR LOF	Defective activation of the classical pathway, diminished clearance of apoptotic cells	SLE, infections with encapsulated organisms	Lupus nephritis
C3 GOF	C3	AD GOF	Hyperactive C3 convertase, activation of alternative complement pathway	aHUS	C3 glomerulopathy, aHUS, thrombotic microangiopathy
Factor B GOF	CFB	AD GOF	Increased formation of C3 convertase, activation of alternative complement pathway	aHUS	C3 glomerulopathy, aHUS
Factor I deficiency	CFI	AR LOF	Reduced CFI level and activity, spontaneous activation of the alternative complement pathway with consumption of C3	Infections, disseminated neisserial infections, aHUS, preeclampsia	C3 glomerulopathy, aHUS
Factor H deficiency	CFH	AD, AR LOF	Impaired inactivation of C3 convertase, activation of alternative complement pathway	Infections, disseminated neisserial infections, aHUS, preeclampsia	C3 glomerulopathy, aHUS
Factor H-related protein deficiencies	CFHR1, CFHR2, CFHR3, CFHR4, CFHR5	AD, AR LOF	Autoantibodies to Factor H, linked deletions of one or more *CFHR* genes, activation of alternative complement pathway	Older-onset aHUS, disseminated neisserial infections	C3 glomerulopathy, aHUS
MCP deficiency	CD46	AD	Inhibitor of complement alternate pathway, decreased C3b binding	aHUS, infections, preeclampsia	aHUS, thrombotic microangiopathy
Defects of *SMARCAL1* gene					
Schimke immuno-osseous dysplasia	SMARCAL1	AR LOF	Defective cellular immunity	Short stature, spondyloepiphyseal dysplasia, intrauterine growth retardation; bacterial, viral, fungal infections; may present as SCID; bone marrow failure	Nephropathy, progressive renal insufficiency, FSGS
Autoinflammatory diseases and AA amyloidosis					
ADA2 deficiency	ADA2	AR LOF	Defects of ADAs deactivating extracellular adenosine and terminating signaling through adenosine receptors; activation of IFN-I pathway	Polyarteritis nodosa, childhood-onset recurrent ischemic stroke and fever; some patients develop hypogammaglobulinemia	AA amyloidosis, thrombotic microangiopathy
FMF	MEFV	AR LOF, AD	Increased pyrin inflammasome-mediated induction of IL-1β	Recurrent fever, serositis and inflammation	AA amyloidosis
MWS	NLRP3	AD GOF	Increased cryopyrin inflammasome-mediated induction of IL-1β	Urticaria, sensorineural hearing loss, amyloidosis	AA amyloidosis
FCAS1	NLRP3	AD GOF	Increased cryopyrin inflammasome-mediated induction of IL-1β	Non-pruritic urticaria, arthritis, chills, fever and leukocytosis after cold exposure	AA amyloidosis
NOMID/CINCA	NLRP3	AD GOF	Increased cryopyrin inflammasome-mediated induction of IL-1β	Neonatal-onset rash, chronic meningitis, and arthropathy with fever and inflammation	AA amyloidosis
TRAPS	TNFRSF1A	AD GOF	Mutations of TNF receptor leading to intracellular receptor retention or diminished soluble cytokine receptor available to bind TNF	Recurrent fever, serositis, rash, and ocular or joint inflammation	AA amyloidosis
MKD (HIDS)	MVK	AR LOF	Deficient/reduced MVK activity, RhoA prenylation inhibition, activation of pyrin inflammasome	Periodic fever and leukocytosis with high IgDlevels	AA amyloidosis
VEXAS syndrome	UBA1	Somatic, LOF	Defective in ubiquitylation, TNF↑, IL-6↑, IFN-γ↑	Late-onset treatment-refractory inflammatory syndrome (fevers, cytopenias, dysplastic bone marrow, interstitial nephritis, chondritis, vasculitis)	AA amyloidosis

a Diseases and key clinical features refer to the 2022 updated classification of inborn errors of immunity compiled by the International Union of Immunological Societies Expert Committee [1].

^b^The disease name of TLR7 GOF mutations refers to the Online Mendelian Inheritance in Man database (https://www.omim.org).

LOF, loss-of-function; GOF, gain-of-function; AR, autosomal recessive; AD, autosomal dominant; IPEX, immunodysregulation, polyendocrinopathy, and enteropathy, X-linked; SLE, systemic lupus erythematosus; IFN-I, type I interferon; AGS, Aicardi–Goutières syndrome; ACL, familial chilblain lupus; GRAD, gain-of-function mutation in RIG-I-associated disease; SAVI, STING-associated vasculopathy with onset in infancy; USP18, ubiquitin-specific protease 18; TLR, toll-like receptor; SPENCD, spondyloenchondrodysplasia; XLR, X-linked recessive; NF-κB, nuclear factor-κB; COPD, chronic obstructive pulmonary disease; ALPS, autoimmune lymphoproliferative syndrome; PFAPA, periodic fever, aphthous stomatitis, pharyngitis, adenitis; IBD, inflammatory bowel disease; ISGs, interferon stimulated genes; JAK/STAT, Janus kinase-signal transducers and activators of transcription; MCP, membrane cofactor protein; SCID, severe combined immune deficiency; FSGS, focal segmental glomerulosclerosis; FCL, familial chilblain lupus; ITP, immune thrombocytopenic purpura; FMF, familial Mediterranean fever; MWS, Muckle-Wells syndrome; FCAS1, familial cold autoinflammatory syndrome 1; NOMID/CINCA, neonatal-onset multisystem inflammatory disease (NOMID) or chronic infantile neurologic cutaneous and articular syndrome (CINCA); TRAPS, TNF receptor-associated periodic syndrome; MKD (HIDS), mevalonate kinase deficiency (Hyper IgD syndrome); VEXAS syndrome, vacuoles, E1 enzyme, X-linked, autoinflammatory, somatic syndrome.

### Diagnosis of PID-caused kidney diseases

Early diagnosis and appropriate treatment of PID are the keys to reducing the mortality rate and improving organ function and quality of life. However, the diagnosis of PID depends on the physician's awareness of the disease and the implementation of strategies to improve the recognition of PID (Table 1).

For a patient suspected with PID-caused kidney disease, a series of diagnostic investigations concerning PID is needed. A stepwise approach for the diagnosis of PID has been recommended [51, 52]. First, infection is a general presentation in PID. Thus, a detailed history of infections should be explored, including the age of onset, site, frequency, organism implicated and therapy needed. For example, any unusual infection, including meningitis, sepsis, fungal or opportunistic organisms, should raise the suspicion of an underlying PID. Second, a general immunological investigation, including a serum immunoglobulin test, complete blood count with differential, lymphocyte subset analysis and complement analysis, is needed. In addition, evaluating specific immune responses is essential for diagnosing of PID. For example, in a patient suspected to have a defect in cellular immunity, flow-cytometry is needed to identify naive and memory T cells, activation markers on T-cell surface and intracellular proteins, and cytokines in different effector T-cell populations. In a patient suspected of having type I interferonopathy, elevated serum interferon-α levels and ISGs overexpression in blood cells are helpful. Specific tests for different PID catalogs have been reviewed recently [3, 51, 53]. Third, genetic testing plays a key role in the diagnosis of PID. Currently, targeted gene panels, whole-exome sequencing and whole-genome sequencing are available for diagnosing and researching PID. In addition, RNA sequencing, especially tissue-specific RNA sequencing, is an invaluable tool for discovering and validating aberrant splicing caused by deep intronic, synonymous and splice-site variants [54]. Establishing a precise genetic diagnosis is desirable for accurate genetic counseling and planning for future pregnancies, defining genotype–phenotype associations and identifying candidates for gene-specific therapies. The concise diagnostic approach is listed in Table 2.

**Table 2:** Key points for the diagnosis of PID-caused kidney diseases.

Warning signs of PID-caused kidney diseases
(i) Early-onset autoimmunity
(ii) A family history of immunodeficiency or immune dysregulation
(iii) Recurrent, unusual, or difficult-to-treat infections (otitis media, sinusitis and pneumonia, diarrhea)
(iv) Multiple immune dysregulation, for instance, autoimmunity combined with recurrent infections, or lymphoproliferative manifestations, or allergy
(v) Presence of autoimmune cytopenia, including autoimmune hemolytic anemia, immune thrombocytopenia, Evan's syndrome, autoimmune neutropenia
(vi) Presence of gastrointestinal symptoms, such as inflammatory bowel disease, nodular regenerative hyperplasia, sclerosing cholangitis
(vii) Presence of multiple endocrine diseases, such as Hashimoto thyroiditis, Graves’ disease, type 1 diabetes, adrenal insufficiency, hypoparathyroidism
A stepwise approach to the diagnosis of PID-caused kidney diseases
(i) Taking a detailed history of infections, including the age of onset, the site, frequency, organism implicated and therapy required
(ii) (a) General immunological investigations, including a serum immunoglobulin test, complete blood count with differential, lymphocyte subset analysis, and complement analysis (b) Evaluation of specific immune responses based on patients’ manifestations, including autoantibody profiles, B-cell maturation testing, T-cell subsets, T-cell proliferative response to mitogens, T-cell cytotoxicity, surface marker expression, cytoplasmic protein phosphorylation and cytokine production (c) *In vitro* radiosensitivity, lipid profiles, enzyme activity assays (d) High-resolution computed tomography scan (lung, brain, abdomen), endoscopy (gastroscopy, colonscopy, thoracoscopy) and tissue biopsy (gut, bone marrow, lymph node, lung)
(iii) Kidney biopsy: the main histological lesions include: (a) inflammatory-proliferative lesions; (b) collapsing glomerulopathy; (c) vascular lesions (TMA, vasculitis); (d) membranous lesions; (e) interstitial nephritis; and (f) AA amyloidosis
(iv) Genetic testing: targeted gene panels, whole-exome sequencing, whole-genome sequencing, RNA sequencing

Importantly, kidney biopsy is a critical diagnostic tool for assessing the pattern of kidney damage in patients with PID-caused kidney diseases. The histological lesions of PID-caused kidney diseases are very heterogeneous and include: (i) inflammatory-proliferative lesions (mesangial, endocapillary, extracapillary and membranoproliferative); (ii) collapsing glomerulopathy; (iii) vascular lesions (TMA, vasculitis); (iv) membranous lesions; (v) interstitial nephritis; and (vi) AA amyloidosis.

### Treatment of PID-caused kidney diseases

The prominent features of PID include immunocompromization, as well as the resulting infection and subsequent autoimmunity/autoinflammation; therefore, treatment strategies include rebuilding immune function, controlling infection and the autoimmune response, and protecting organs from damage [52, 53].

Immunoglobulin replacement is the main treatment for the prevention of recurrent infections and is indicated for all disorders with significantly impaired antibody production. Commonly utilized agents to manage autoimmunity in PID include glucocorticoids, anti-CD20 antibodies and specific T-cell immunosuppressants. The regimens of immunosuppression for autoimmunity in PID may refer to the corresponding autoimmune disease without PID. However, overimmunosuppression should be avoided because of the increased susceptibility to infections in these patients. In the case of lupus nephritis caused by PID, for example, a regimen of reduced-dose glucocorticoids following a short course of methylprednisolone pulses may be considered when both the kidney and extrarenal disease manifestations show satisfactory improvement.

**Figure 2:**
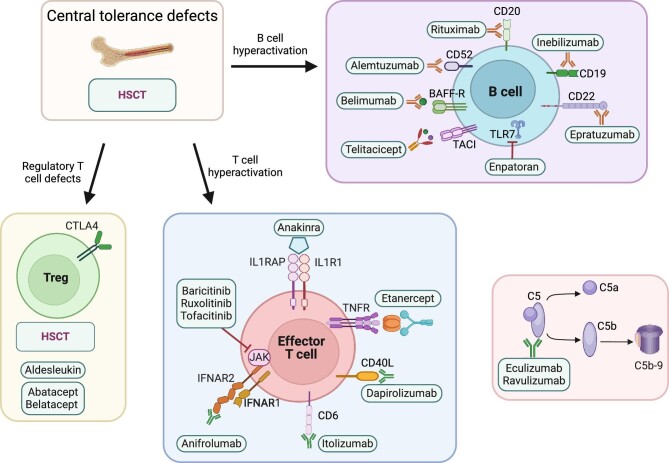
Potential therapeutic targets in PID-caused kidney diseases. Recent emerging therapies based on different targets are indicated. Abatacept, CTLA4-immunoglobulin fusion protein; aldesleukin, recombinant human IL-2; alemtuzumab, humanized anti-CD52 monoclonal antibody; anakinra, IL-1 receptor antagonist; anifrolumab, anti-IFN-α receptor monoclonal antibody; baricitinib, JAK1 and JAK2 inhibitor; belatacept, CTLA4-immunoglobulin fusion protein; belimumab, BAFF-specific monoclonal antibody; dapirolizumab, CD40 ligand antagonist; eculizumab, humanized anti-C5 monoclonal antibody; enpatoran, toll-like receptor 7 inhibitor; epratuzumab, humanized anti-CD22 monoclonal antibody; etanercept, tumor necrosis factor-α inhibitor; inebilizumab, humanized anti-CD19 monoclonal antibody; itolizumab, humanized anti-CD6 monoclonal antibody; ravulizumab, humanized anti-C5 monoclonal antibody; rituximab, CD20-specific monoclonal antibody; ruxolitinib, JAK1 and JAK2 inhibitor; telitacicept, TACI-Fc fusion protein targeting BAFF and a proliferating-inducing ligand (APRIL); tofacitinib, JAK1 and JAK3 inhibitor. Abbreviations: CTLA4, cytotoxic T lymphocyte-associated antigen 4; HSCT, hematopoietic stem cell transplantation; IFNAR1, type I interferon receptor 1; IFNAR2, type I interferon receptor 2; TNFR, tumor necrosis factor receptor; IL1R1, interleukin-1 receptor type 1; IL1RAP, interleukin-1 receptor accessory protein; BAFF, B cell activating factor; BAFF-R, BAFF receptor; TACI, transmembrane activator and calcium-modulator and cyclophilin ligand (CAML) interactor; JAK, Janus kinase. Created with BioRender.com.

In recent years, pathway-based therapeutic approaches have been implemented to improve the clinical condition of patients with PID (Fig. 2). Targeted or precision therapy represents a new area in which medical treatment is tailored based on the affected gene or pathway. This reinforces the importance of genetic testing for identifying specific mutations in each patient to precisely diagnose and properly manage patients.

#### JAK inhibitors

JAK inhibitors are small molecules that inhibit the signal transduction through the JAK/STAT pathway. JAK inhibitors have been successfully used in treating several type I interferonopathies, such as DNase II deficiency, Aicardi–Goutières syndrome and familial chilblain lupus [55–57]. Most patients showed significant clinical improvement in terms of infections, autoimmune manifestations, skin lesions or lymphoproliferation. Only a few studies on the effectiveness of JAK inhibitors in PID-caused kidney diseases are available. Tofacitinib, ruxolitinib and baricitinib have been used in patients with kidney diseases caused by *IFIH1, STING, DDX58* or *SOCS1* mutations, and improvements in proteinuria, hypocomplementemia and kidney abnormalities were reported [21, 33, 36, 58].

#### Monoclonal antibodies targeting the interferon-α receptor

Anifrolumab is a fully human IgG1κ monoclonal antibody targeting interferon-α receptor 1 that was approved for the treatment of SLE by the US Food and Drug Administration. Moreover, anifrolumab in combination with standard therapy improved the complete renal response in patients with active, biopsy-proven, class III/IV lupus nephritis [59]. There are no reports on the treatment of various PIDs with anifrolumab. However, given the importance of type I interferon for triggering autoimmunity in PID-caused kidney diseases, targeting the type I interferon pathway is a potential therapeutic strategy.

#### TLR7 inhibitors

Enpatoran is a potential small molecule that blocks the activation of TLR7. Given the importance of TLR7 signaling in driving human lupus, phase II clinical trials evaluating the efficacy and safety of orally administered enpatoran in SLE and cutaneous lupus erythematosus have been initiated (NCT05162586, NCT05540327). The efficacy of TLR7 inhibition for PID and PID-caused kidney diseases warrants further study.

#### Cytokine inhibitors of the NF-κB pathway

Given that genetic defects in the NF-κB pathway promote immune-mediated kidney diseases, NF-κB pathway-targeted therapeutic strategies might be applied clinically. For example, tumor necrosis factor (TNF)-α and IL-1 are activators and effectors of the NF-κB pathway. The TNF-α inhibitor etanercept and the IL-1 receptor antagonist anakinra markedly suppress NF-κB activation and have been successfully used in patients with lupus nephritis caused by *TNFAIP3* mutations [60, 61]. However, further clinical studies are needed to assess the benefits of such biologics in more kidney disease patients with genetic defects in the NF-κB pathway.

#### Complement inhibitors

Eculizumab is a humanized monoclonal antibody that prevents the cleavage of the human complement of C5 and subsequent formation of the MAC. It is very effective in the treatment of paroxysmal nocturnal hemoglobinuria and aHUS, as it can prevent the progression to end-stage kidney disease. Eculizumab is also effective in the treatment of lupus nephritis patients with TMA harboring rare variants or polymorphisms in complement genes [49].

#### Stem cell transplantation

Most PIDs are caused by genetic defects in hematopoietic cells. Therefore, replacement of mutant cells with healthy donor hematopoietic stem cells represents a rational therapeutic approach. Hematopoietic stem cell transplantation (HSCT) has been applied to treat a broader range of PIDs. For instance, refractory early-onset autoimmune disease due to a *TNFAIP3* LOF mutation was successfully treated with HSCT [62]. There are few reports of HSCT for PID-caused kidney diseases. Two patients with kidney diseases due to FOXP3 mutations were treated with HSCT. One presenting with diarrhea, eczema, hypothyroidism, interstitial nephritis and autoimmune hemolytic anemia was transplanted with a matched sibling donor at 10 years of age. He achieved remission and long-term survival. The other presenting with tubulointerstitial nephritis, food allergy, diabetes, and diarrhea was transplanted with a mismatched related donor at 7 years of age. However, the patient developed grade III graft-versus-host disease and soon died due to microangiopathy and kidney failure [17]. A personalized approach to HSCT for PID may limit therapeutic side effects and promote robust long-term immune reconstitution and quality of life.

## CONCLUSION

Genetic defects that trigger immune dysregulation are major causes of PID-caused kidney diseases. The diverse clinical presentations of PID pose diagnostic challenges for clinicians; thus, clinician awareness is needed for the detection of these diseases. An extensive understanding of the mechanisms underlying PID-caused kidney diseases contributes to the choice of genetic screening and individual treatment. Early initiation of targeted therapy may improve the overall outcomes of these patients. Pathway-based therapy, HSCT and gene therapy have introduced a broad field to pursue novel therapies for patients with PID. By exploiting medical genetics and therapeutics, PID-caused kidney diseases are becoming diagnosable and treatable diseases.

## Data Availability

Not applicable. All the data are included in the manuscript itself.
